# Identification of the Genomic Insertion Site of Pmel-1 TCR α and β Transgenes by Next-Generation Sequencing

**DOI:** 10.1371/journal.pone.0096650

**Published:** 2014-05-14

**Authors:** Yun Ji, Natalie Abrams, Wei Zhu, Eddie Salinas, Zhiya Yu, Douglas C. Palmer, Parthav Jailwala, Zulmarie Franco, Rahul Roychoudhuri, Eric Stahlberg, Luca Gattinoni, Nicholas P. Restifo

**Affiliations:** 1 Experimental Transplantation and Immunology Branch, Center for Cancer Research, National Cancer Institute, National Institutes of Health, Bethesda, Maryland, United States of America; 2 Advanced Biomedical Computing Center, Frederick National Laboratory for Cancer Research, Frederick, Maryland, United States of America; 3 Surgery Branch, Center for Cancer Research, National Cancer Institute, National Institutes of Health, Bethesda, Maryland, United States of America; National University of Singapore, Singapore

## Abstract

The pmel-1 T cell receptor transgenic mouse has been extensively employed as an ideal model system to study the mechanisms of tumor immunology, CD8^+^ T cell differentiation, autoimmunity and adoptive immunotherapy. The ‘zygosity’ of the transgene affects the transgene expression levels and may compromise optimal breeding scheme design. However, the integration sites for the pmel-1 mouse have remained uncharacterized. This is also true for many other commonly used transgenic mice created before the modern era of rapid and inexpensive next-generation sequencing. Here, we show that whole genome sequencing can be used to determine the exact pmel-1 genomic integration site, even with relatively ‘shallow’ (8X) coverage. The results were used to develop a validated polymerase chain reaction-based genotyping assay. For the first time, we provide a quick and convenient polymerase chain reaction method to determine the dosage of pmel-1 transgene for this freely and publically available mouse resource. We also demonstrate that next-generation sequencing provides a feasible approach for mapping foreign DNA integration sites, even when information of the original vector sequences is only partially known.

## Introduction

Transgenic animal models are indispensable resources for studies of gene function and disease. Their construction often involves large bacterial or yeast artificial chromosomes, which are used to assemble the transgene [1]. Unfortunately, many transgenic lines remain poorly characterized, and the method for generating these transgenic animals (i.e. the injection of genetic material into the pro-nucleus of a fertilized egg) results in the random integration of foreign transgenic DNA into the genome [1]. Generally transgenic animals are commonly evaluated by Southern blot to determine gene incorporation [1]. Southern blotting can also provide a rough estimate of copy number, but does not indicate zygosity. The site of integration, the possibility of rearrangements of the transgene and potential deletions of non-lethal native DNA at the site of integration remain unknown for most transgenic lines. It is worth noting here that transgenes may tend to integrate into sites of active gene transcription. Furthermore, many non-lethal sites of integration can disrupt or alter the function of genes that although non-lethal but key to physiologic functions [1]. Thus, random and unknown integration of transgenes can affect the behavior of transgenic mice in unpredictable ways [1].

To minimize ‘unknown unknowns’ and to design optimal breeding schemes or evaluate dosage effects of transgenic animals, it is necessary to identify the site of integration, which is generally only one, and also distinguish heterozygotes from homozygotes because zygosity can affect the behavior of transgenic mice. Ideally, the method employed need to be rapid and easy. Fluorescence in situ hybridization (FISH) [2], as currently practiced, is not as ‘high throughput’ or as straightforward as polymerase chain reaction (PCR) to determine zygosity of transgenic lines as it is slower, more expensive and labor-intensive.

PCR provides an optimal and reliable assay for the purpose of managing mouse breeding colonies, but is only feasible if the genomic integration site is known. Various methods have been currently employed to identify the integration sites of foreign DNA fragments in the genome. Among them, inverse PCR is the most commonly used but its feasibility is limited unless optimal restriction enzyme for digesting the inserted fragment is available [3]. Splinkerette PCR is another approach, which was originally developed to amplify the genomic DNA between a known restriction site and a target gene [4], and later adapted to map the insertion sites of retroviral integrating sites in the mouse genome [5]. Alternatively, a more conventional method could be employed by multiple steps of cloning and sub-cloning plus FISH, Southern blot, library construction, screening and sequencing [6]. All together, these methods are very laborious and cost-ineffective, seriously limiting the progress of mapping transgenic insertion sites.

The pmel-1 mouse was developed as a model system for studying the treatment of malignant melanoma using adoptive cell therapy [7]. The target antigen, pmel-17, is an ortholog of the melanocyte differentiation antigen gp100, which is often overexpressed in human melanomas [8]. Adoptive transfer of transgenic T cells expressing the gp100-specific T cell receptor (TCR) from pmel-1 mice can effectively mediate the regression of large established tumors when administered in combination with a lymphodepleting preconditioning regimen [9]–[13], exogenous γc cytokine [7], [9], [14]–[18], and vaccination with the cognate antigen [7], [15], [19], [20]. Pmel-1 mouse now is available from the Jackson laboratory (http://www.jax.org).

Recent clinical success of T cell-based cancer immunotherapy [21]–[27] has resulted in a surge in demand for the pmel-1 mouse as an ideal model system to study the mechanism of tumor immunology, CD8^+^ T cell differentiation, and adoptive immunotherapy [28]–[59]. Moreover, pmel-1 mice have been increasingly employed to study autoimmune vitiligo [7], [60]–[64] and uveitis [61], [63], [65]. The pmel-1 mouse was generated using two transgenic vectors, which contained variable domains of the endogenous TCR as described previously [7] (Figure S1). To design an efficient breeding scheme with other transgenes, it is essential to identify the zygosity status of the pmel-1 transgene. However, the highly identical and repetitive nature of transgenic TCR α and β chains with the endogenous loci and the large size of the construction vectors made it especially daunting to determine the integration site with established methods. Currently, determination of whether a mouse that is genotypically positive for the pmel-1 transgene is homozygous or heterozygous relies on observation of Mendelian inheritance rules, which is a time-consuming and labor-intensive process.

Here we propose an alternative strategy to identify pmel-1 genomic integration site(s) based on shallow next-generation sequencing (NGS) of the entire transgenic mouse genome. Recently, a transgenic insertion site was identified by using a combination of microarray hybrid capture and NGS analysis [66]. This approach can be very useful when the sequence of a transgene is known, which is not applicable for the pmel-1 case. In this study, we applied the NGS to analyze the pmel-1 genome and successfully identified the integration site of pmel-1 TCR α and β transgenes. This information allowed us to design PCR assay to easily distinguish heterozygous from homozygous animals. The results obtained by PCR were consistent with previous observation that pmel-1 homozygous or heterozygous CD8^+^ T cells can be distinguished by different tetramer staining efficiency, confirming the results obtained from the NGS data analysis.

## Methods

### Ethics statement

The care and use of all mice in this study was carried out with the approval of the National Cancer Institute Animal Use and Care Committee (protocol # SB-126).

### Mice

Pmel-1 (B6.Cg-*Thy1^a^*/Cy Tg(TcraTcrb)8Rest/J) were obtained from the Jackson Laboratory.

### Antibodies and flow cytometry

We purchased all FACS antibodies from BD Biosciences Flow cytometry acquisition was performed on a BD FACSCanto Ι or BD FACSCanto ΙΙ flow cytometer. Samples were analyzed with FlowJo software (Tree Star).

### Genomic DNA sample preparation

Genomic DNA (gDNA) was extracted from pmel-1 homozygous transgenic animal tail with OmniPrep for Tissue (G BIOSCIENCES) following the manufacturer's instruction. Genomic DNA was analyzed by nanodrop and agarose gel to verify the quality (O.D. 260/280 ratio is more than 1.8) and quantity (1 ug for library construction).

### Illumina library preparation and sequencing

Sequencing was done on Illumina HiSeq 2000 using TruSeq v3 chemistry at the Center for Cancer Research sequencing facility, SAIC-Frederick/FNLCR, Frederick, MD. The gDNA sample was sequenced on one individual lane with paired-end read length of 101 bp. Raw data was processed and basecalling performed using the standard Illumina pipeline (CASAVA version 1.8.2 and RTA version 1.8.70.0).

### Illumina data quality control

Over three hundred million (2*157,554,381) pass-filter reads were generated. Quality control analysis of the sequencing reads was conducted using the in-house Illumina QC 08.08.2012 workflow, which is based on FastQC (www.bioinformatics.babraham.ac.uk/projects/fastqc) and Picard (picard.sourceforge.net) software tools. The description of the workflow is available at http://ccrifx.cancer.gov/apps/site/workflows_for_bioinformatics_analysis. Over 84% of all pass-filter reads had a Basecall quality score above 30, while library complexity was over 81% for Pmel-1 libraries. Mapping to reference mouse genome (mm9) has shown that more than 75% read aligned uniquely with low mismatch error rate (1–1.2%).

### Illumina read mapping and analysis

Reference-guided assembly was performed using Bowtie [67], [68]. To detect discordant read pairs, all reads that passed the standard Illumina chastity filter were aligned to the mouse nuclear and mitochondrial genomes (mm9, http://genome.ucsc.edu) using Bowtie version 2.0.0-beta7 and allowing for two mismatches per read (bowtie2-2.0.0-beta7/bowtie2 —un un.txt —al al.txt —un-conc unconc.txt —al-conc alconc.txt —met-file mets.txt -x genome -1 read_unconc.1.fastq -2 read_unconc.2.fastq -S map.sam). The “—un-conc " option was used to capture read pairs that could not be aligned to the reference genome concordantly. These discordantly mapped read pairs (unconc.txt) were passed on to SVDetect and DELLY for structural variant detection.

In parallel, we also used Bowtie to detect soft-clipped reads associated with the insertion site. This time Bowtie was used in a "local" alignment mode (–local), which doesn't require that reads align end-to-end (bowtie2-2.0.0-beta7/bowtie2 —un un.txt —al al.txt —un-conc unconc.txt —al-conc alconc.txt —met-file mets.txt -x genome -1 read_R1.fastq -2 read_R2_all.fastq -S map.sam). This way the reads were "soft-clipped" at one or both ends to optimize alignment score and allow for local alignment. This approach identified several subsets of “split reads”, which had either both ends mapped to different genomic locations or only a portion of the read mapped to the genome reference.

### Structural variation detection using DELLY

We used two DELLY modules, DELLY-DUPS and DELLY-JUMPY using default parameters. DELLY-DUPS extracts data from a reference sequence file and a sequence alignment (BAM) file and outputs filtered lists of predicted structural variations (SVs), their genomic coordinates, and confidence scores as a tab-separated text. DELLY-DUPs runs yielded hundreds of candidate duplication sites, which were captured in a VCF (Variant Call Format) file. This candidate SVs were filtered by supporting evidence filters (read quality and genotype) and then ranked by normalized high-quality read count ("RC") for each predicted duplication variant. After excluding the first two candidates associated with mitochondrial chromosomes and repeats, the top candidate duplication was placed near a TCR region on chromosome 14. Because the reads were mapped to the reference genome, these "duplications" appear to be located on chromosomes 6 and 14, in the vicinity of TCR regions. DELLY-JUMPY generated hundreds of candidate translocations that were filtered by average mapping quality and then ranked by the number of supporting split-reads and by the location identified by DELLY-DUPS.

### SVDetect analysis of chromosome-spanning read pairs

Quality-filtered discordant (spanning) read pairs were analyzed using SVDetect to identify regions associated with structural rearrangements (SRs). The software was run at default setting. Top-scoring SVDetect-filtered read pairs were reviewed and filtered manually to remove: SRs with too few or many supporting reads (fewer than 5 or larger than 16); SRs mapping to centromeres or the mitochondrial chromosome; SRs with multiple adjacent rearrangements. The insertion site boundaries were supported by eight read pairs spanning chromosomes 2 and 6 and five pairs spanning chromosomes 2 and 14. The exact insertion site was confirmed by the presence of split reads that were soft-clipped at one or both ends to optimize alignment score and allow for local alignment.

### Genotyping PCR

Genomic DNA was extracted with QuickExtract DNA extraction solution 1.0 (EPICENTRE). PCR was performed under standard conditions. Primers used were Ch2Pmel-F1: ctttagacctccggcactgttgc; Ch2Pmel-R1: gcaagtagcagtgtatcaaatatgc; PmelTCR-R1: gtagctttgtaaggctgtggagag. Ch2Pmel-F1 and Ch2Pmel-R1 amplify a transgenic band of 308 bp. Ch2Pmel-F1 and PmelTCR-R1 amplify an endogenous band of 203 bp.

## Results

### Tetramer analysis distinguishes the zygosity status of pmel-1 mice

**Figure 1:**
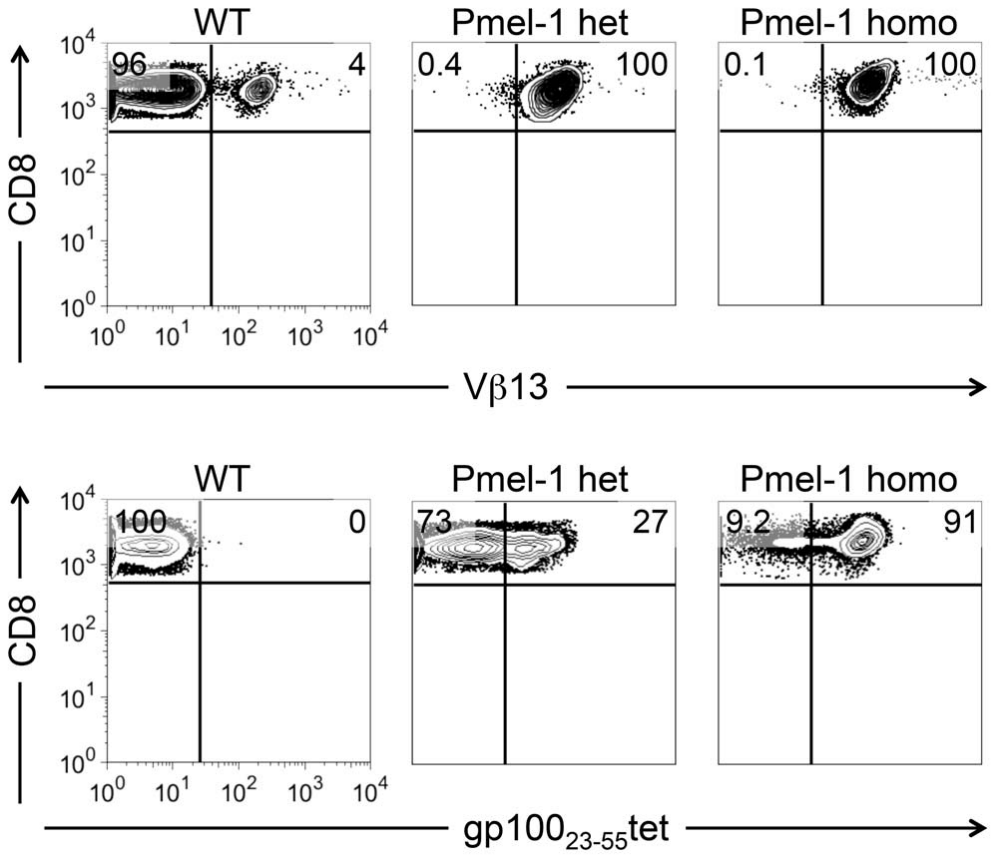
Pmel-1 TCR tetramer staining distinguishes the gene dosage of pmel-1 transgene in CD8^+^ T cells. Staining of pmel-1 heterozygous or homozygous CD8^+^ T cells with antibodies recognizing pmel-1 TCR β chain Vβ13 (upper panel) or tetramers recognizing pmel-1 TCR (lower panel).

Currently the identification of the zygosity status of pmel-1 transgene is based on deducing the breeder genotype by Mendelian inheritance rules. Interestingly, we discovered that gp100 D^b^ tetramer staining enable to distinguish pmel-1 homozygous from heterozygous CD8^+^ T cells. There were no notable differences of intensity or frequency between homozygous and heterozygous cells stained with the antibody specific for the Vβ13 chain of pmel-1 TCR (Figure 1, upper panel). However, both frequency and intensity of tetramer^+^ T cells were reduced in heterozygous compared to homozygous pmel-1 T cells (Figure 1, lower panel). Although this observation allow us to distinguish pmel-1 homozygosity or heterozygosity, the requirements of FACS analysis of naïve CD8^+^ T cells, the cost of tetramers, and the inability to perform this type of assay reliably on neonatal mice limit the application of this method.

### Determination of the pmel-1 integration site by low-coverage whole genome sequencing

We performed shallow NGS of the pmel-1 homozygous mouse, which produced 8X coverage of the genome, to identify the transgenic insertion sites. Since the pmel-1 transgene contained multiple tandem copies of mouse endogenous TCR domains [7], we used this information to streamline our analysis strategy. Notably, knowing the exact integrative cassette sequence was not a prerequisite for the identification of the integration site. Although this partial knowledge of the transgene origin has facilitated the validation of the integration site.

**Figure 2:**
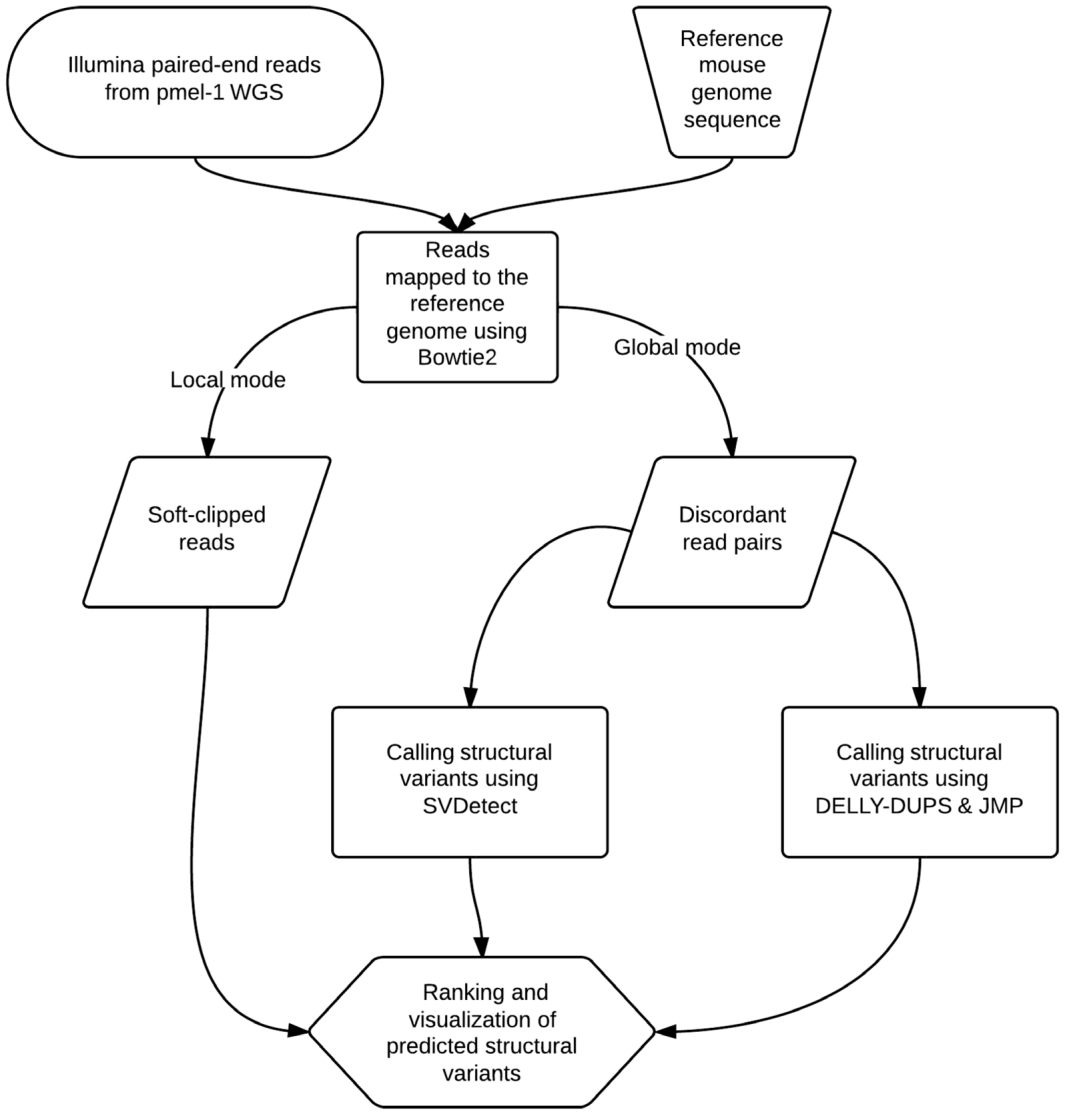
NGS data analysis workflow. This chart depicts the analysis of NGS data from Whole Genome Sequencing. In this analysis, standard tools for small structural variation detection were used to predict large insertions based on short paired-end (2*100 bp) sequence reads. Both SVDetect and DELLY extract data from a reference sequence file and a sequence alignment (BAM) file and generate filtered lists of predicted structural variations, their genomic coordinates, and confidence scores as a tab-separated text. Downstream steps, such as data visualization, cross-sectioning and ranking of SVs were done in the Integrative Genomics Viewer and Excel.

To identify candidate integration site, we searched for evidence of structural variations such as tandem duplications and translocations based on discordant NGS reads pairs. To that end, we first aligned paired-end Illumina reads from whole-genome sequencing to the reference mouse genome using Bowtie in global (reads align end to end) and local modes (reads are soft-clipped) (Figure 2). From the global alignment, only uniquely and discordantly mapped read pairs (4,092,067 reads or 2.6%) were retained for the subsequent analyses. These read pairs were analyzed further using the SVDetect [69] and DELLY (PMID: 22962449) software to identify potential structural variants (SVs). These SV candidates were filtered to remove predictions associated with repeats, mitochondria, centromeres, and intra-chromosomal rearrangements and then ranked based on confidence scores. After filtering, the top candidate duplication was placed near a TCR region on chromosome 14, while a duplication associated with a TCR region on chromosome 6 was ranked 23^rd^.

These duplications appear to be located on chromosomes 6 and 14 because sequence reads were mapped to the reference genome, which does not have tandem copies of TCR domains. These findings confirmed our expectations and provided us with way to identify junction read pairs bridging these regions and the area surrounding the yet unknown insertion site. Since we anticipated that in pmel-1 both regions immediately adjacent to the insertion site might have been derived from endogenous TCR regions on chromosomes 6 and 14, our next goal was to find these discordant read pairs that aligned partially to these TCR loci.

**Figure 3:**
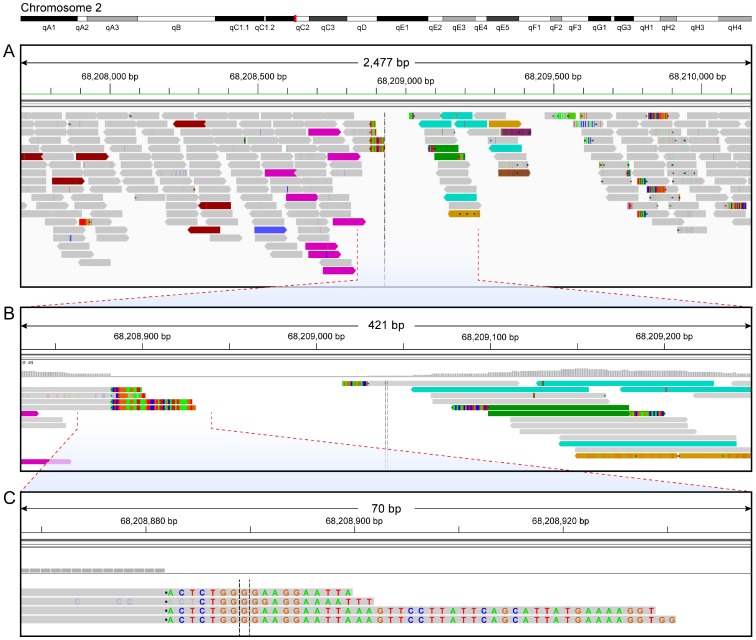
Read alignments around the insertion site of pmel-1. An ideogram of chromosome 2 is shown at the top with the red bar indicating the area of insertion. Gray bars represent concordant read pairs. Red and blue colors represent read pairs where the insert size is larger than expected (deletion) or smaller than expected (insertion), respectively. Discordantly mapped reads are coded by the chromosome on which their mates can be found, so reads represented by magenta and aquamarine rectangles have mates that mapped to chromosomes 6 and 14, respectively. Other colors represent genomic “noise”. Multicolored blocks represent misaligned areas within reads. Soft-clipped reads were represented by grey bar with multicolored blocks at the ends. Misaligned bases in soft-clipped reads are shown in blue (C), green (A), red (T) and orange (G) color, respectively. (**A**) A snap shot of pmel-1 sequence reads aligned to the reference mouse genome near the integration area. Eight magenta-colored discordant reads are on the left side and five aquamarine-colored are on the right side. (**B**) An enlarged view of pmel-1 sequence reads, which shows five soft-clipped reads, one on right side, four on left sides of the insertion area. (**C**) A nucleotide level view of pmel-1 reads near the insertion area. This panel shows four soft-clipped reads that mapped partially to the β chain on chromosome 6 and partially to chromosome 2 of the reference genome.

These filters narrowed thousands of SVDetect and DELLY-JUMPY predictions down to several candidate structural variants supported by discordant junction read pairs. Subsequently we used these junction read pairs as well as soft-clipped reads generated by local alignment to identify the exact genomic coordinates of the integration site. This step was done by manually inspecting local alignment files using the Integrative Genomics Viewer (IGV) [70], [71]. Our results showed that the top candidate insertion site was supported by 13 discordant read pairs and 5 soft-clipped reads (Figure 3). In Figure 3A, these reads flank the area in the reference genome that corresponds to the insertion site in the pmel-1 genome. Figure 3B shows five junction reads that aligned partially to chromosome 2 and partially to α or β chain regions on chromosomes 14 and 6, which harbor the respective endogenous TCR loci in the reference genome. The insertion site is located in the 3′UTR region of the gene Stk39, which encodes a serine/threonine kinase that may function in the cellular stress response pathway [72].

The pmel-1 insertion is accompanied by a 157-bp deletion (chr2: 68,208,872–68,209,029 bp). Such deletions are common in transgenic mice [73], [74]. The left side insertion boundary (chr2: 68,208,872 bp) was supported by 8 discordant read pairs and 4 soft-clipped reads mapped partially to the β sequence and partially to chromosome 2 (Figure 3B, 3C). In contrast, the right-side boundary was supported by 5 discordant read pairs and 1 soft-clipped read aligned with the α sequence and chromosome 2, consistent with insertion at 68,209,029 bp (Figure 3B). The insertion area is bounded by a 5-bp duplicated sequence (TGGAT) on the right and by a 7-bp duplicated sequence (CCAGCAG) on the left, which are identical between the regions on chromosome 2 and the α and β TCR sequences. Such homologous regions are thought to facilitate chromosomal rearrangements that accompany a transgene insertion.

### Confirmation of the integration sites by PCR analysis

**Figure 4:**
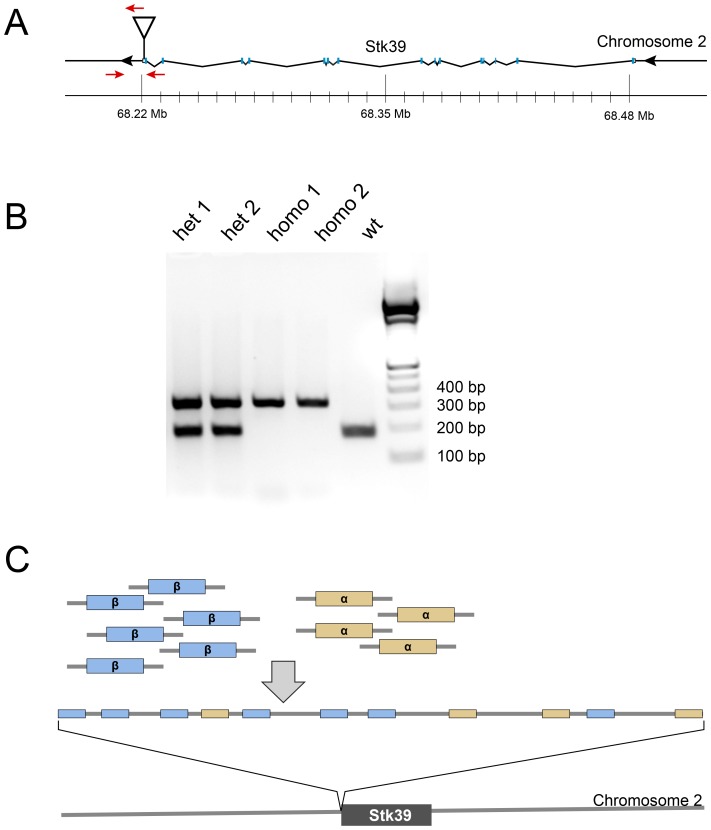
PCR analysis distinguishes the homozygosity or heterozygosity of pmel-1 transgene. (**A**) Diagram of PCR primers designed from the predicted pmel-1 TCR β transgene insertion site on chromosome 2. (**B**) PCR results of pmel-1 homozygous or heterozygous animals. (**C**) Conceptual model of tandem integration of the pmel-1 α and β chains in the genome.

The identification of the potential integration site of TCR β chain at 68,208,872 bp on chromosome 2 allows us to design three primers for PCR genotyping analysis, which are located at the 5′ and 3′ of the integration site, and also at the 3′ of the integrated pmel-1 TCR β chain transgenic vector junction site, respectively (Figure 4A). According to the design, we would observe a single 308 bp band from the PCR products of homozygous pmel-1 transgenic animals and a single 203 bp band from wild type animals while both the 203 bp and the 308 bp bands would appear from heterozygous animals. Consistent to our expectations, we observed distinctive PCR bands with the exact predicted size (Figure 4B). Furthermore, the transgene dosage determined by the PCR analysis was consistent to the conclusions drawn from the tetramer staining analysis (Figure 1), further corroborating that the integration site resolved by whole-genome sequencing data analysis was correct.

## Discussion

In this study, we identified the genomic integration site of pmel-1 TCR α and β transgenes by performing the NGS of pmel-1 genome. Based on the predicted integration site, we further designed a fast and convenient PCR method to distinguish pmel-1 transgene dosage. The conclusions made from PCR genotyping assay are consistent with the results obtained by FACS analysis of pmel-1 T cells and prediction based on Mendelian inheritance rules, confirming the validity of the integration sites identified by NGS analysis.

The alignment results are consistent with a pattern of tandem integration of the vectors for both pmel-1 TCR α chain and β chain. To estimate how many copies of α and β sequences were inserted into each transgenic animal genome, we calculated the mean coverage for the entire genome as well as α and β regions on chromosomes 14 and 6, respectively. The genome coverage was estimated as 8X, while the insert copy numbers are 16X and 4X for α and β cassettes, respectively. Except for a few gaps, most vector sequences appear to be integrated into the pmel-1 genome. Therefore, we proposed a model for the potential layout of the transgene in the genome (Figure 4C).

The identification of pmel-1 genomic integration site will not only greatly facilitate the genotyping for researchers employing the pmel-1 model system, but also allow a more efficient and effective breeding scheme design. Many researchers breed pmel-1 transgenic mouse with other transgenic strains to evaluate the function of other transgenes that are involved or potentially involved in T cell development or function. However, previously all the breeding schemes were designed blindly and the certainty of obtaining desired progeny is susceptible to risk. The identification of the insertion site can also prevent futile attempts to breed pmel-1 with transgenes knockout animals where the relevant gene of interest is actually located very close to the pmel-1 transgene on chromosome 2. Thus, identification of the integration site can prevent unnecessary losses of time and resources.

In our system we have demonstrated the possibility of identification of transgene integration site with little information of the original design of transgenic animals. In principle, with the advances in whole genome sequencing technology, this method can be applied to characterize all other transgenic animals [66]. Furthermore, NGS data of the genomic DNA can be further used for genome comparison, another alternative choice for animal genetic background test besides microsatellite test. In this study, we focused on the identification of the pmel-1 integration site, but NGS can also indicate details about the genetic background of the mice, such as their similarity to other mice on the ‘C57BL/6’ background. This information can be useful to understand mechanisms underlying immune rejection as well as host and donor compatibility. The sequences for pmel-1 genomic DNA have been deposited to NCBI Short Read Archives (SRA) and free for the public access, which will provide significant value to the transgenic mice genome studies.

### Data Visualization

The Integrative Genomics Viewer (IGV) [70], [71] and SAMtools were used for data visualization [75].

### Data Access

Read sequence data has been submitted to the NCBI Short Read Archive (SRA) under accession number SRP037973 and BioProject accession No. PRJNA238124.

## Supporting Information

Figure S1 **Diagrams of original constructs for generating pmel-1 transgenic animals.** (A) Pmel-1 TCR α transgenic vector. The variable region of TCR α were cloned between Xmal and Not I sites. (B) Pmel-1 TCR β transgenic vector. The variable region of TCR β were cloned between Xho I and Sac II sites.
(TIF)
