# Supplementary figures and images for: Identification of the Genomic Insertion Site of Pmel-1 TCR α and β Transgenes by Next-Generation Sequencing
Source: PLoS One. 2014 May 14;9(5):e96650. doi: 10.1371/journal.pone.0096650 (PMC4020793; doi:10.1371/journal.pone.0096650)

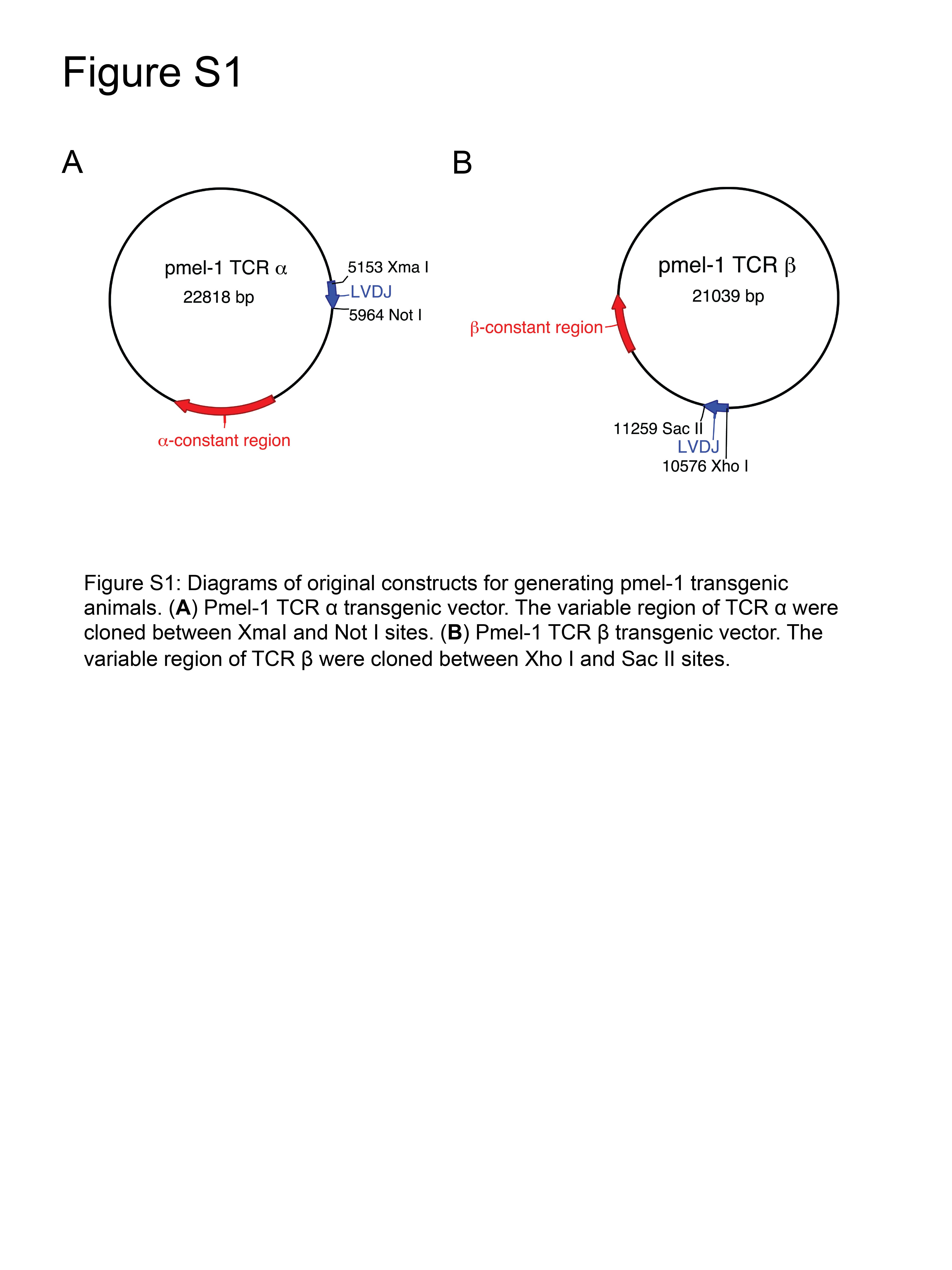

Supplement: Figure S1 — Diagrams of original constructs for generating pmel-1 transgenic animals. (A) Pmel-1 TCR α transgenic vector. The variable region of TCR α were cloned between Xmal and Not I sites. (B) Pmel-1 TCR β transgenic vector. The variable region of TCR β were cloned between Xho I and Sac II sites. (TIF) [file pone.0096650.s001.tif]
